# Triple-target radiosurgery for intractable cancer pain of mixed origin: Two-centre experience in Central America

**DOI:** 10.1177/20494637251350331

**Published:** 2025-06-18

**Authors:** Paola Del Cid, Liliana Aquino, Alejandra Moreira, Víctor Caceros, Carlos Tobar, Alejandro Blanco, Gabriel Carvajal, Luis Bermudez-Guzman, Eduardo E. Lovo

**Affiliations:** 1 Radiosurgery, International Cancer Center, Diagnostic Hospital, San Salvador, El Salvador; 2 Robotic Radiosurgery Center, RinggoldID:92690 International Cancer Center, San José, Costa Rica

**Keywords:** Cancer pain, Triple-target radiosurgery, Palliative care, Neuromodulation

## Abstract

Cancer pain is one of the most severe components of the symptom burden among cancer patients, especially those with advanced or metastatic disease. Palliative interventions are necessary to alleviate cancer pain and reduce opioid-related side effects, thereby minimizing patient suffering. Radiosurgery has been effectively used to target the medial thalamus and the hypophysis for the treatment of chronic pain syndromes. These two areas are critical for pain modulation and control, and their precise targeting with radiosurgery and its non-invasive nature can provide relief for patients suffering from cancer-related intractable pain. Our previous work with single target irradiation of the hypophysis revealed promising pain relief in terminal cancer patients, albeit more suited for hormone-mediated tumours or bone-derived pain rather than complex mixed pain syndromes. Given that, we previously introduced the concept of triple-target irradiation (hypophysis + both thalami) in a small report of terminally ill cancer patients. Here, we report a larger case series of terminally ill patients (*n* = 8) with complex cancer pain treated with a triple-target approach, with radiation doses generally considered low or non-ablative (90 Gy), in contrast to the usual single-target, ablative approach comprising higher doses. We noted a substantial decrease in VAS scores and the medications needed to manage pain across all patients, experiencing minimal to no side effects. Our findings indicate that a minimally invasive triple-target method, utilising low radiation doses, effectively alleviates pain, lowers medication dependency, and enhances the quality of life with few side effects. Furthermore, additional research is essential to optimise pain relief and ensure long-term effectiveness.

## Introduction

Pain is widely acknowledged as one of the most severe components of symptom burden among cancer patients, particularly those with advanced or metastatic disease. Such pain can be a consequence of the tumour itself or secondary to therapeutic procedures and tends to worsen with chronicity. Additionally, chronic cancer-related pain can be exacerbated by unpredictable episodes of breakthrough pain in around 31%–45% of patients.^1^ By late-stage disease, 76% of patients report moderate to severe pain, often undermanaged, and typically caused by a combination of nociceptive and neuropathic mechanisms. In about 10%–30% of cases, patients become unresponsive to standard analgesic treatment.^2,3^

End-of-life symptoms and cancer-related pain are treated mostly with opioids. Since medication and substance use disorders have steadily increased worldwide in recent years, this class of medications, long used by oncologists, is now being scrutinised and restricted.^4^ Due to tighter regulatory dispenser rules in the face of an opioid crisis, addiction concerns lead to an undertreatment of cancer pain among cancer patients. Therefore, palliative interventions to mitigate cancer pain and reduce opioid requirements are necessary to reduce patient suffering and opioid-induced side effects.^5^ Other factors such as socioeconomic status, access to medical care, inflammation, or the nature of the cancer and its treatment may further contribute to inadequate pain management.

Stereotactic radiosurgery (SRS) has historically been used for the management of pain (malignant and non-malignant) classically through single target irradiation of the medial thalamus or hypophysis.^6^ Lars Leksell pioneered the use of SRS in 1968 to target substructures of the medial thalamus in two patients suffering from malignancy-related intractable pain.^7^ Some years later, radiosurgical hypophysectomy was also established for the treatment of cancer pain.^8^ Today, this treatment is considered safer than invasive measures and relieves a significant percentage of patients within a few hours or days.

Pituitary SRS is particularly effective for patients with cancer-related pain, achieving a success rate (defined as a reduction in pain by >50%) of 87%, whereas thalamic SRS is more effective for patients with non-malignant pain, with a success rate of 65%.^9^ The exact mechanisms involved are unclear, but it is suggested that a neuromodulatory response to radiation is responsible for the fast treatment response and the frequent lack of visible lesions following the hypophysectomy.

We have previously reported the use of single-target, dual-target, and triple-target irradiation for malignant and non-malignant pain management.^10–13^ Following a single-target approach, we have observed pain relief after irradiation of the hypophysis.^10^ However, this approach may be better suited for hormone-mediated tumours or bone-derived pain rather than mixed pain syndromes involving abdominal, pelvic, or neuropathic pain. We have previously reported the preliminary results in pain relief using a triple-target approach combining hypophysectomy and bilateral thalamotomy with a small series of three patients.^13^ We hereby continue that initial series with a larger series of terminally ill patients (*n* = 8) with complex cancer pain (including the three previously reported patients) being treated with a triple-target approach with radiation doses generally considered as low or non-ablative, in contrast to the usual single-target, ablative approach.

## Methods

Patients were treated with Infini® (Masep Medical Company, Shenzhen, China) in our centre in El Salvador or Cyberknife® robotic radiosurgery system (Accuray Inc. California) in our centre in Costa Rica. Under local anaesthesia, a neurosurgeon placed an Infini® stereotactic frame (Masep Medical Company, Shenzhen, China) on patients treated with this system. For Cyberknife® patients, a thermoplastic mask (Klarity R450U, 2.4 mm) was used as an immobiliser system before the acquisition of the images.

Magnetic Resonance Images were acquired with a 1.5-Tesla Avanto® (Siemens Corporation, Erlangen, Germany) for Infini® patients. T1-weighted and T2-weighted images were obtained (1 mm slice thickness) with no spacing covering the thalamus until the superior border of the corpus callosum. Computed tomography (CT optima 560, GE Medical Systems) was obtained for Cyberknife® patients (1.25 mm slice thickness) without intravenous contrast medium. Magnetic resonance images (Signa Explorer, GE Medical Systems) were also acquired in T1 sequences with and without GD and FIESTA, which are co-registered with the computed tomography image for the delineation of both treatment volume and organs at risk.

Images were imported into the Multiplan® and Superplan® systems accordingly. For the thalamic targets, the anterior commissure and posterior commissure (PC) were identified in the axial plane, then the images were transformed into the sagittal plane using fusion tools and the intercommissural line (ICL) was drawn between T1 and T2 sequences. The distance from the PC was taken anteriorly along the ICL (usually 4 mm) and confirmed and adjusted by taking 8 mm posterior to the midpoint of the ICL; this was identified as Y. A 90° angle was traced from the PC to the Y along the ICL, and Z was determined 3 to 4 mm above the ICL. Finally, images were reoriented to axial views, and X coordinates were 4 to 6 mm lateral from the contralateral thalamic border to the side of the pain.

**Figure 1.**
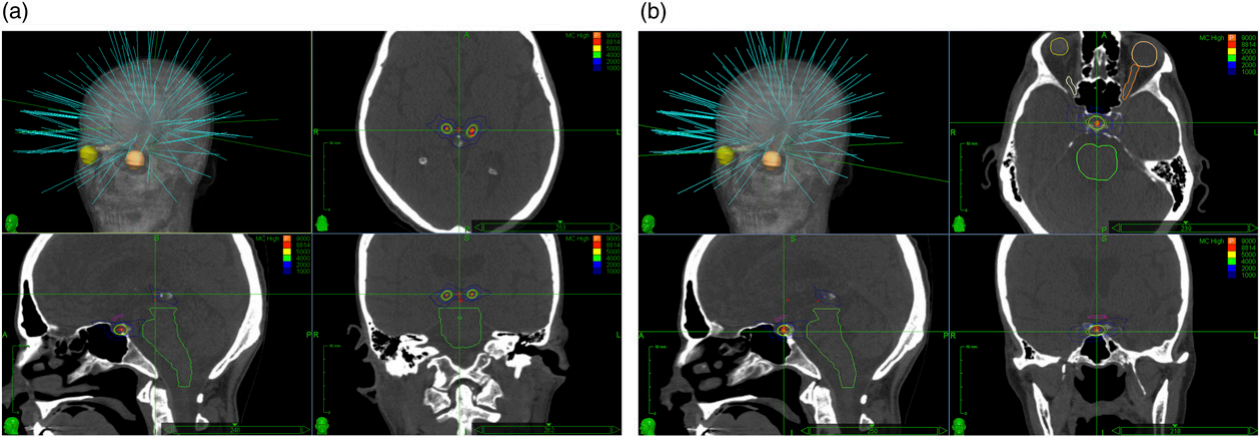
Representative treatment plan for the triple-target approach using CyberKnife stereotactic radiosurgery. (a) 3D reconstruction and representative CT scans in axial, sagittal, and coronal sections for the irradiation of both medial thalami. (b) 3D reconstruction and representative CT scans in axial, sagittal, and coronal sections for the irradiation of the hypophysis. Dose conformation is represented by the coloured isodose lines, ranging from 20% to 100% of the dose (in cGy, where the thick orange line is the prescription isodose, the green isodose curve represents 40 Gy and the most external isodose line corresponds to 10 Gy in Cyberknife.

The prescribed dose of 90 Gy was delivered to the medial regions of the thalamus and the hypophysis (Figure 1), with the isodose set at 100%. For the hypophysis, the neurohypophysis was defined as the isocenter of the shot. The treatment was administered in a single session, using a fixed collimator system of 4 mm for thalamus and 8 mm for hypophysis. Dose restrictions of high-risk organs were respected for a single session, as described by the literature. We assessed patients’ health status using the EQ-5D-5L questionnaire before treatment and at the final follow-up, while the VAS score was recorded at multiple time points post-treatment (24 h, 1 week, 2 weeks, and the last follow-up). Additionally, we retrospectively analysed the pain medication prescribed by the algology specialists, who were not involved in this study. This analysis reflects the clinical decisions made independently by the treating physicians based on each patient’s condition, ensuring that any changes in medication use were driven by patient needs rather than study influence.

## Results

From February 2022 to October 2022, eight patients (three males and five females; average age: 51.4 years; range 18–74) were referred to both of our centres and treated using our triple-target approach (Table 1). All were terminally ill patients with various types of cancer, most of whom had metastatic disease and had exhausted all available treatment options for their primary cancer, with no expectation of recovery. All patients were on palliative care and required multiple medications for their pain, combining opioids with NSAIDs and anticonvulsant neuromodulators such as gabapentin, local anaesthetic, palliative chemotherapy, and percutaneous procedures (Table 1). Two patients required an epidural elastomeric morphine pump, and one of them also required supraclavicular and epidural catheters with lone local anaesthetic or combined with analgesics (Bupivacaine 1.5–2 mg), respectively. Most patients reported a Visual Analogue Scale (VAS) of 10 before treatment (mean = 9.6, SD = 0.74).

**Table 1.** Patient demographics and characteristics.

Patient	Sex	Age	Primary disease	Metastases	Primary treatment	Duration of disease	Type of pain	Location of pain	Pain management	Treatment platform	Follow-up (days)
1	M	65	Gastric adenocarcinoma	Lungs, lumbar vertebrae	T11 vertebroplasty + radiofrequency + RT	4 months	Mixed	Thorax, lower back, hips, lower limbs	CTx + Opioids + NSAIDs	GK	21
2	F	18	Recurrent Ewing’s sarcoma	None	Surgical resection	3 years	Somatic, neuropathic	Shoulder, arm, left thoracic wall	Opioids + Local anaesthetic + BTX nerve block of the brachial plexus	CK	29
3	M	66	Pancreatic cancer (stage IV)	None	N/D	N/D	Mixed	Chronic abdominal pain, muscle pain in the neck, shoulders, back, hips and lower limbs	Celiac plexus neurolysis (no response) + opioids + NSAIDs + neuromodulator + local anaesthetic	CK	30
4	M	74	Epidermoid skin carcinoma	Axillary nodes	Surgical rescue	N/D	Somatic, neuropathic	Right hemicrania and surgical site	NSAIDs + opioids + neuromodulator	CK	107
5	F	35	Cervical cancer	Bone metastases in hip and T8-T12 vertebral bodies	N/D	N/D	Visceral	Headache, constant pain in the lower limb	Opioids + local anaesthetic + CTx	CK	100
6	F	34	Breast cancer	Cerebellar metastases, bone metastases in the vertebral body and left pedicle of L4	CTx + RT + Surgical resection	7 years	Somatic, neuropathic	Lower back and lower limb	Opioids	GK	73
7	F	53	Right breast cancer + right brachial plexopathy	Ipsilateral axillary adenopathy	CTx + RT + surgical resection	2 years	Mixed	Upper limb	Opioids	GK	41
8	F	66	Thigh leiomyosarcoma	Multiple bone metastases	Surgical resection + RT	1 year	Somatic, neuropathic	Lower limb	NSAIDs + opioids + neuromodulator	GK	38

*Note.* GK: GammaKnife; CK: CyberKnife; CTx: Chemotherapy; RT: Radiotherapy; NSAIDs: Non-steroidal anti-inflammatory drugs; N/D: no data.

**Figure 2.**
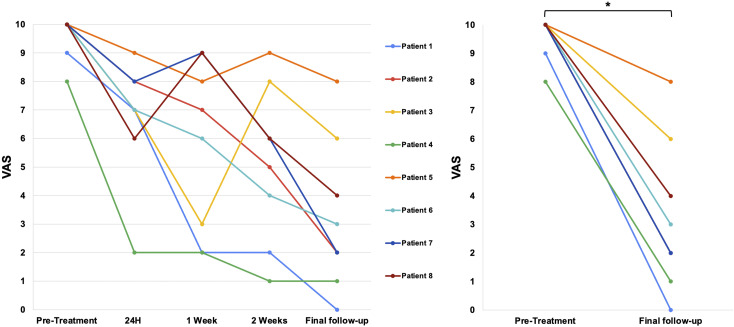
Evolution of pain intensity according to Visual Analogue Scale (VAS) scores. Right panel: Wilcoxon Signed-Rank test, p = 0.014.

**Figure 3.**
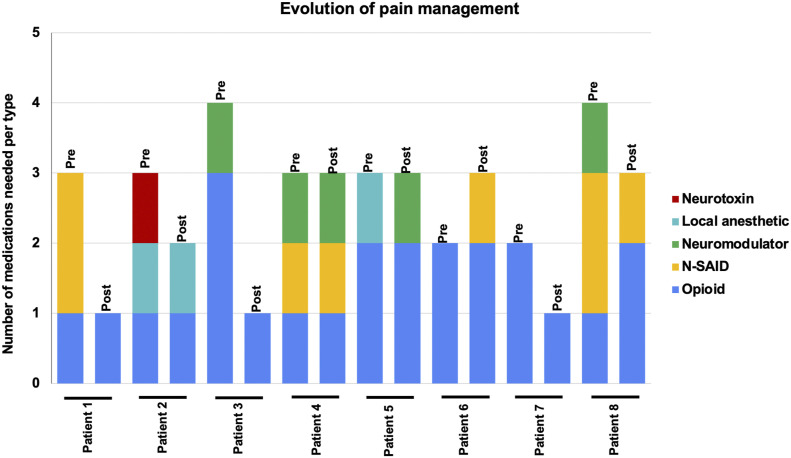
Evolution of pain management according to the number of medications needed. “Pre” corresponds to before treatment and “Post” corresponds to the last follow-up. Neurotoxin corresponds to botulinum toxin.

Following treatment, we obtained the VAS score after 24 h, 1 week, 2 weeks, and at the last follow-up (before death). All patients experienced a degree of pain relief 24 h after treatment, and all of them died with a significantly lower VAS score (Figure 2, Wilcoxon Signed-Rank test, *p* = 0.014). The mean VAS Score went from 9.6 pretreatment to 6.7 (SD = 2.5) after 24 h, 5.2 (SD = 2.0) after 2 weeks, 3.8 (SD = 3.1) after 30 days, and 4.2 (SD = 2.6) at the last follow-up. Moreover, we observed a notable reduction in the number of pain medications required by most patients, aligning with decreased VAS scores and indicating a positive treatment response (Figure 3). There were several changes in medication use during the follow-up time. Five of the eight patients were able to reduce their opioid requirements, either by lowering their dosage or by managing their pain with a single opioid. Three patients presented worsening pain due to complications of their primary disease and required opioid management. Specifically, one patient suffered pulmonary atelectasis, and two patients suffered urinary tract infections. At the final follow-up, six patients reported improved responses to opioid medication, with both patients and caregivers noting greater pain relief from the same opioid dosage compared to the relief experienced before SRS. One patient who was able to reduce opioid usage during the first week presented a brief episode of dizziness and blurred vision secondary to chemotherapy.

**Figure 4.**
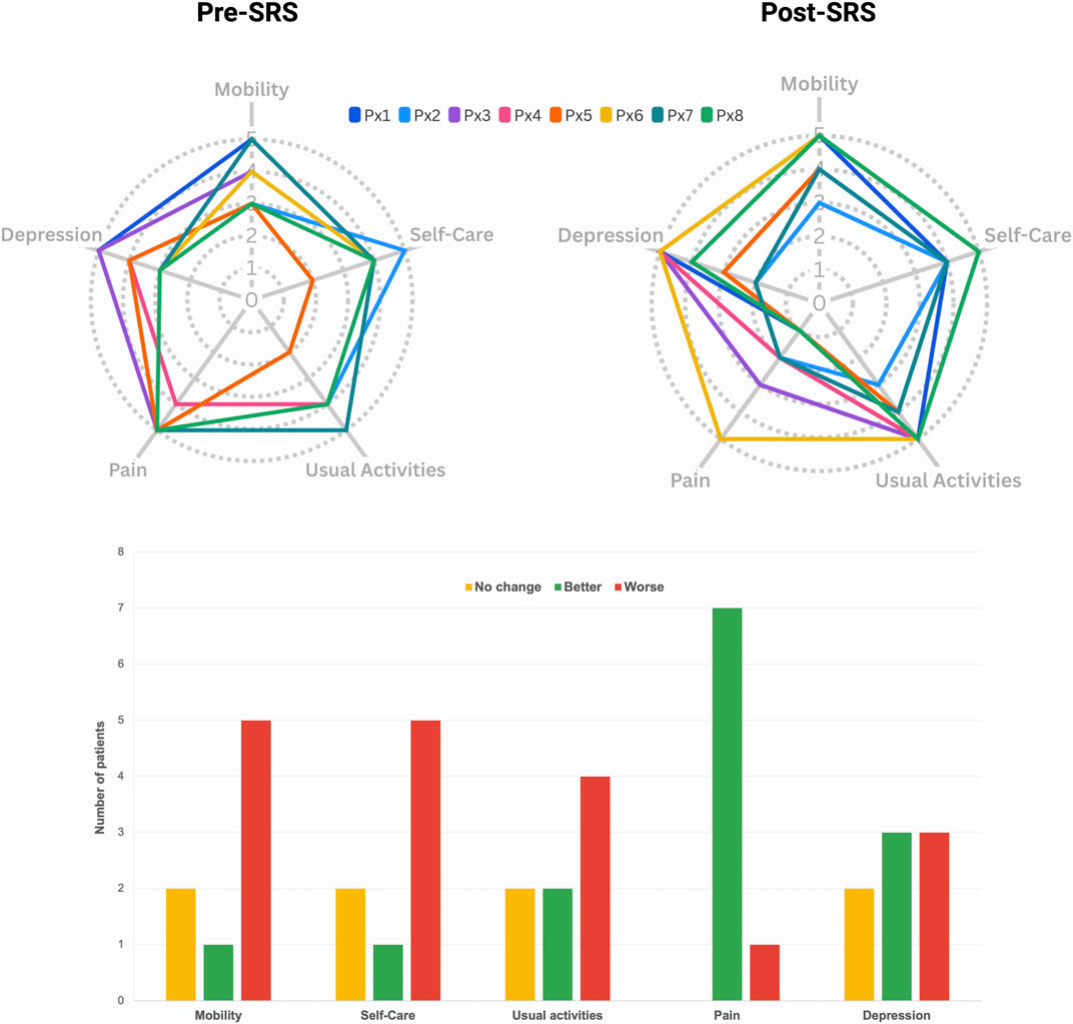
Changes in quality-of-life measures across dimensions. Top: Radar plots display changes in each dimension before and after treatment for each patient, with “Post-SRS” representing the last follow-up. Bottom: Bar chart showing the number of patients who experienced no change, improvement, or worsening in their condition for each quality-of-life dimension post-treatment.

Additionally, we used the EQ-5D-5L questionnaire to evaluate the five dimensions of health in our patients. We observed changes across all five dimensions of the EQ-5D-5L (mobility, self-care, usual activities, pain/discomfort, and anxiety/depression). Notably, each dimension had at least one patient reporting improvement and at least one reporting deterioration post-treatment. The most significant positive change was observed in the pain/discomfort dimension, where 7 out of 8 patients reported improvement, and only one experienced a decline (Figure 4). Given the terminal nature of the patient’s conditions, it is likely that observed deteriorations in certain health dimensions are attributable to disease progression rather than the treatment itself. The procedure was well tolerated by all patients, with only one patient reporting headache and another reporting fatigue in the first 24 h after treatment. Other acute effects of either bilateral thalamotomy or hypophysectomy were not reported by the patients.

## Discussion

Around 75% of late-stage cancer patients suffer from cancer pain that may be difficult to manage using pharmacological strategies, and surgery can become a risky option. In such cases, radiosurgery can be a safer and more effective approach to alleviate pain during terminal stages and improve the quality of life for both patients and their caretakers. The use of surgical hypophysectomy, while extremely effective for pain relief, has been hindered by the endocrine damage produced by surgical approaches. However, the development of radiosurgical techniques has provided a much safer option with the same efficacy.^14^

This case series examined a novel triple-target approach (hypophysis and both thalami) for managing complex cancer pain in terminally ill patients, using lower, non-ablative radiation doses compared to the traditional single-target, high-dose method. All patients showed significant reductions in VAS pain scores and medication requirements, with minimal to no side effects. Our findings highlight the potential of stereotactic radiosurgery (SRS) as an effective intervention for oncologic pain management. While changes were observed across all EQ-5D-5L dimensions, the most notable improvement was in pain/discomfort, with seven out of eight patients reporting relief. Although some patients experienced declines in other health dimensions, these are likely attributable to disease progression rather than the treatment itself. This suggests that SRS may offer meaningful pain relief even in patients with advanced illness, reinforcing its role as a valuable palliative option.

Radiosurgical hypophysectomy may selectively inhibit chronic, cancer-related pain via C-fiber afferents while allowing acute pain transmission through A-fiber afferents.^5^ Evidence suggests gamma hypophysectomy stimulates rather than damages the pituitary gland, challenging the term “hypophysectomy” as there is no clinical data indicating gland destruction.^15^ Supporting this, patients show no endocrine dysfunction or MRI abnormalities post-treatment, with clinical symptoms and MR spectroscopy indicating increased hypothalamic neuron activity within 24 h.^16^ Unlike thalamic pain syndrome, where pain often recurs within a year, cancer pain patients are less likely to experience recurrence.^14,15,17^ The medial thalamus, particularly the centromedian-parafascicular complex (CM-Pf) within the intralaminar nuclei, is a key target for cancer pain syndromes, as it plays a central role in regulating the affective components of pain.^18^

High-dose irradiation (140–250 Gy) targeting medial thalamic structures like the CM-Pf complex often provides immediate pain relief, with 55% of patients reporting positive effects, though relief is typically short-lived, particularly in mixed pain syndromes, and recurrence is common in longer-surviving patients.^19^ This rapid relief suggests a potential neuromodulatory effect of radiation (radiomodulation) rather than tissue destruction.^20^ Due to the short duration of pain relief, medial thalamotomy alone is generally insufficient for managing cancer pain. While hypophysectomy alone initially relieves pain in 80% of cases, relief is variable, with 50% of patients experiencing severe pain by the end of life.^10^

For patients with limited life expectancy and high symptom burden, achieving early and lasting pain relief is critical for effective symptom palliation. Combining irradiation of both medial thalami and the hypophysis may provide more durable relief by addressing both pain transmission and its emotional impact. Our previous case report on a triple-target approach—90 Gy to the hypophysis and 120 Gy to the medial thalami—demonstrated significant pain reduction within 72 h, lasting until death, and reduced opioid usage by 70%–84% in all patients.^13^ This low-dose approach contrasts with conventional higher doses (140–180 Gy) and may activate radiomodulatory effects rather than destructive outcomes. Additionally, targeting the periaqueductal grey could involve broader pain regulation pathways.

For mixed pain syndromes, subablative doses (60–100 Gy) can potentially provide durable pain relief by altering neural circuits without causing cell loss, through radiomodulation.^21,22^ In this context, the present study applies a multitarget, low-dose approach (90 Gy to the hypophysis and both thalami) aiming for effective pain management via radiomodulation. While not fully subablative, these doses may initially slow neuronal transmission, resulting in durable pain relief. The 45–20 Gy isodose lines could modulate areas within the mediodorsal (MD), ventroposterolateral (VPL), and ventroposteromedial (VPM) thalamic nuclei, which process pain signals to the limbic system—regions known for altered activity in chronic cancer pain, such as the insula, anterior cingulate cortex (ACC), and prefrontal cortex (PFC).^23,24^

Radiosurgical hypophysectomy appears to be safer than chemical or surgical approaches, with minimal risk of serious side effects when doses remain below 200 Gy.^5^ Although higher doses in other studies caused significant complications, medial thalamic lesioning at lower doses did not result in motor, sensory, or cognitive deficits.^19,25,26^

Further studies with larger sample sizes, control arms, and post-stereotactic radiosurgery (SRS) imaging are recommended to validate efficacy and ensure long-term safety. Despite limitations, such as short patient survival and lack of imaging data, this novel approach shows promise as a palliative treatment for advanced cancer pain.

## Conclusions

Terminally ill cancer patients are often afflicted by a mix of several pain syndromes for which traditional and even invasive methods of analgesia are often insufficient, resulting in considerable patient suffering and diminished quality of life for patients and carers. A multitarget, minimally invasive approach using radiosurgery might represent a more suitable option for the interacting mechanisms of chronic cancer-related pain afflicting these patients. Additionally, providing faster, safer, and long-lasting relief significantly eases the burden of terminal illness for patients and carers. The current case series reports the use of the lowest doses of radiation with a triple-target approach, achieving a significant reduction in VAS scores and the number of medications, with minimal to no side effects. While no current strategy can translate into complete elimination of pain, a multitarget approach with lower doses can turn an unmanageable mixed pain syndrome into a more responsive, bearable one.
